# Metabolic syndrome is independently associated with a mildly reduced estimated glomerular filtration rate: a cross-sectional study

**DOI:** 10.1186/s12882-017-0597-3

**Published:** 2017-06-13

**Authors:** Wen Hu, Xiao-Juan Wu, Yao-Jun Ni, Hai-Rong Hao, Wei-Nan Yu, Hong-Wen Zhou

**Affiliations:** 1 Department of Endocrinology, The First Affiliated Hospital of Nanjing Medical University, Nanjing, Jiangsu 210029 China; 2 Department of Endocrinology and Metabolism, Huai’an Hospital Affiliated to Xuzhou Medical University and Huai’an Second People’s Hospital, Huai’an, 223001 China; 3 Department of Cardiothoracic Surgery, Hospital Affiliated to Nanjing Medical University and Huai’an First People’s Hospital, Huai’an, 223001 China; 4 Department of Endocrinology, The First Affiliated Hospital of Nanjing Medical University, No. 300 Guangzhou Road, Nanjing, 210029 China

**Keywords:** Metabolic syndrome, Mildly reduced eGFR, Hyperfiltration, Chinese, Renal damage

## Abstract

**Background:**

Association between metabolic syndrome (MS) and mildly reduced estimated glomerular filtration rates (eGFRs) remains unclear. Therefore, we aimed to evaluate the association between MS and a mildly reduced eGFR in Chinese adults.

**Methods:**

Anthropometric and biochemical examinations were performed in 2992 individuals. The eGFR was calculated from the creatinine level. MS was defined according to the Adult Treatment Panel III criteria as the presence of three or more risk factors. Mildly reduced eGFR was defined as a value between 60 and 90 mL/min/1.73 m^2^. Multiple linear regression and multiple logistic regression analysis were used to evaluate association between metabolic syndrome and estimate glomerular filtration rate.

**Results:**

After adjusting for several potential confounders, the participants with MS showed a 1.29-fold increased odds ratio for a mildly reduced eGFR compared with those without MS. Additionally, the odds ratios (and 95% confidence intervals (CIs)) for mildly reduced eGFR in participants with elevated triglycerides (TG), decreased high-density lipoprotein (HDL), obesity and elevated fasting blood glucose (FPG) after multivariable adjustment were 1.25 (1.05–1.49), 1.23 (1.03–1.48), 1.22 (1.03–1.45) and 0.64 (0.52–0.78), respectively. The odds ratios (95% CIs) for hyperfiltration in participants with elevated FPG and HbA1c levels after multivariable adjustment were 1.53 (1.30–1.81) and 2.86 (2.00–4.09), respectively.

**Conclusions:**

MS is associated with an increased risk of a mildly reduced eGFR in the Chinese population, and several individual components of MS have different impacts on eGFR levels. MS had dual roles on renal damage.

**Trial registration:**

ChiCTR-TRC-14005029. Registered 28 July 2014.

**Electronic supplementary material:**

The online version of this article (doi:10.1186/s12882-017-0597-3) contains supplementary material, which is available to authorized users.

## Background

Chronic kidney disease (CKD), which is characterized by albuminuria or reduced kidney function, is a worldwide public health problem with increasing incidence and prevalence [1, 2]. It has been demonstrated that patients with CKD have an increased risk of cardiovascular events and death [3, 4]. Furthermore, even mild renal insufficiency increases the risk of cardiovascular events [5, 6] and also serves as a predictor of the progression of kidney disease [7]. Therefore, the early detection of CKD, which is characterized by a mildly reduced estimated glomerular filtration rate (eGFR) and other contributing risk factors, is of critical importance.

A series of abnormalities, including hypertension, dyslipidemia, abdominal obesity, and insulin resistance, indicate the presence of metabolic syndrome (MS). MS has been associated with cardiovascular disease, stroke, and all-cause mortality in the general population [8, 9], and epidemiologic observations have suggested an independent association between MS and CKD [10–12]. Song et al. reported that among a total of 75,468 participants, there was a clear relationship between MS and a reduced GFR, with an odds ratio (95% CI) of 1.43 (1.13–1.83) [13]. Participants without MS showed higher levels of urinary albumin excretion, a lower GFR, and a greater prevalence of CKD, even after adjusting for age and gender [14]. However, most studies have focused on cases of MS and severe kidney diseases where in the eGFR was less than 60 mL/min/1.73 m^2^, and few studies have been conducted to determine the association between MS and a mildly reduced eGFR defined as a value between 60 and 90 mL/min/1.73 m^2^. Therefore, the associations between MS and mildly reduced eGFR have not yet been elucidated.

In the present study, our objective was to evaluate the association between MS and eGFR in a cross-sectional study of middle-aged and elderly Chinese adults. The primary objectives were to examine the relationship between the individual elements of MS and a mildly reduced eGFR for improving preventive and therapeutic effects.

## Methods

### Study population

Objectives in this study were from the Huai’an diabetes prevention program (HADPP) (ChiCTR-TRC-14005029). This program enrolled 10,000 retired workers who had physical exanimation in the Second People’s Hospital from June 2014 to February 2015, who were treated with diet and exercise management. After three-year follow-up, incidence, hospitalization rate and complications of DM were analyzed. This study enrolled only 4249 objectives to conduct a cross section analysis. A total of 4249 participants, ranging from 40 to 79 years of age, in the framework of routine health examinations were recruited from August to October 2014. Participants with the following were excluded: 1) missing data for calculating the eGFR (*n* = 566); 2) previously diagnosed kidney disease, including nephritis, autoimmune or drug-induced kidney disease, renal failure, or kidney transplant with dialysis treatment (*n* = 59); 3) previously diagnosed serious hepatic disease, including fatty liver, liver cirrhosis and autoimmune hepatitis (*n* = 232); 4) peripheral arterial sclerosis disease (*n* = 27); 5) coronary heart disease (CHD), including myocardial infarction and angina pectoris (*n* = 256); or 6) an eGFR <60 mL/min/1.73 m^2^ (*n* = 117). Finally, a total of 2992 participants (1933 women) were eligible for the analysis.

### Data collection

The demographic characteristics, lifestyle information and medical history were obtained by trained investigators through a standard questionnaire. Body mass index (BMI) was calculated as the subject’s weight (kg) divided by their height squared (m^2^). Blood pressure (BP) was consecutively measured 3 times (OMRON Model HEM-752 FUZZY, Omron Company, Dalian city, Liaoning Province, China). After an overnight fast, venous blood samples were collected for measurement of fasting blood glucose (FPG), and postprandial blood glucose (PBG) was measured after participants had completed a 75-g oral glucose tolerance test (OGTT). The creatinine and lipid profiles (total cholesterol (TC), triglycerides (TG), low-density lipoprotein-C (LDL-C) and high-density lipoprotein-C (HDL-C)) and the HbA1c levels were measured using high-performance liquid chromatography (VARIANT II and D-10 Systems, BIO-RAD, Hercules, CA, USA). The eGFR was calculated from the creatinine levels using the Chronic Kidney Disease Epidemiology Collaboration (CKD-EPI) formula [15].

### Definitions

GFR classifications: A normal eGFR (NGFR) was defined as ≥90 mL/min/1.73 m^2^ besides the participants with hyperfiltration, and renal hyperfiltration was defined as GFR >90th percentile after adjusting for sex, age, weight, height, and the use of ACE inhibitors or ARB. This was done by selecting all participants >90th percentile in the distribution of residuals from a multiple linear regression analysis where we used absolute GFR as a dependent variable and sex, age, weight, height and use of ACE inhibitors or ARB as independent variables [16]. A mildly reduced eGFR (MRGFR) was defined as a value between 60 and 90 mL/min/1.73 m^2^.

The presence of MS was defined according to the Adult Treatment Panel III (ATP III) criteria as the presence of three or more of the following risk factors [17]: 1) obesity: a waist circumference > 90 cm in men and >80 cm in women; 2) elevated TG: a serum TG level > 1.70 mmol/L (150 mg/dL); 3) reduced HDL-C: an HDL-C level < 1.04 mmol/L (40 mg/dl) in men or <1.30 mmol/L (50 mg/dl) in women; 4) elevated BP: BP >130/85 mmHg and/or the use of antihypertensive medications; and 5) elevated FPG: a serum glucose level > 6.11 mmol/L (110 mg/dl) and/or the use of insulin or hypoglycemic medication.

The presence of diabetes was defined according to the 2012 American Diabetes Association (ADA) criteria as follows [18]: FPG ≥126 mg/dL (7.0 mmol/L) or 2-h plasma glucose in the 75-g OGTT ≥200 mg/dL (11.1 mmol/L) or HbA1c ≥6.5%. Control of diabetes criteria: HbA1c < 7.0%. Control of diabetes: HbA1c < 7.0%. Control of hypertension criteria [19]: SBP/DBP <140/90 mmHg. Sodium in taking criteria: >2300 mg/day.

### Statistical analysis

The continuous variables in this study, which included a large cohort of people, exhibited a normal distribution or an approximately normal distribution and are presented as the means ± SD, and the categorical variables are presented as numbers (%). The differences between the groups were analyzed using one-way analysis of variance (ANOVA), with non-parametric tests for non-normally distributed variables and chi-square test for categorical data. After verifying the assumption of a linear relationship between the dependent and independent variables that were introduced into the linear regression model, a multiple linear regression analysis was used to estimate the association of the MS and/or its components with the eGFR. A logistic regression analysis was used to assess the association of MS and/or its components with the prevalence of a mildly reduced eGFR. We constructed unadjusted and multivariable-adjusted models (incorporating age and gender, drinking, smoking, hypertension, control of diabetes and hypertension and use of ACE inhibitors or ARB) (Additional file 1: Table S1). In this study, the proportion of patients using other antihypertensive drugs or antihyperlipidimic agents was small, which is shown clearly in Additional file 1: Table S1. The odds ratios and 95% confidence intervals (CIs) were calculated using exponentiation of the logistic regression coefficients. Moreover, to analyze the relationship between MS and hyperfiltration, we performed a logistic regression analysis using the same dependent variables and models for hyperfiltration. A *P* value <0.05 was considered statistically significant. All of the statistical analyses were performed using SPSS 16.0 (SPSS Inc., Chicago, IL, USA).

## Results

### Characteristics of participants

A total of 117 participants with an eGFR <60 mL/min/1.73 m^2^ were excluded. Thus, 2992 participants were enrolled in the study (1933 females aged 40–79 years, 1059 males aged 40–79 years) and divided into three groups according to the eGFR classification criteria in definitions. As shown in Table 1, nearly all of the characteristics evaluated differed among the three groups, with the exception of the percentage of individuals with diabetes, systolic, diastolic BP and cause of MS. As the GFR increased, age, BMI, waist circumference, TC, TG, LDL-C, serum uric acid, serum creatinine, blood urea nitrogen (BUN), the number of males, the prevalence of smoking and drinking, and a history of hypertension were significantly decreased, whereas the FPG, HbA1c and HDL-C levels were significantly increased (all *P* < 0.001). Moreover, the percentage of individuals with 0–2 MS components was higher in the groups with a GFR ≥90 mL/min/1.73 m^2^ compared with those with a GFR between 60 and 90 mL/min/1.73 m^2^, whereas the percentage of individuals with 3–5 MS components was lower. Accordingly, the percentage of participants with MS components was significantly higher among participants with a mildly reduced GFR. By contrast, participants with hyperfiltration were more likely to have elevated FPG levels than those with normal and reduced eGFRs. Patients were divided into two groups according to eGFR: one group with eGFR between 60 and 75 and one group with eGFR between 76 and 89. Compared with eGFR 60–75 group, age, BMI, waist circumference, TC, TG, LDL-C, serum uric acid, serum creatinine, BUN, the number of males, the prevalence of smoking and drinking, and a history of hypertension decreased significantly, while the FPG, and HDL-C levels increased significantly in eGFR 76–89 group. In eGFR 60–75 group, the number of patients with MS components more than 3 was significantly more than that in eGFR 76–89 group.

**Table 1:** Characteristics of the study participants

	eGFR classifications			
Characteristics	MRGFR (*n* = 1479)	NGFR (*n* = 1213)	Hyperfiltration (*n* = 300)	*P*-value
Male, n (%)	640 (43.3)	297 (30.5)	122 (40.7)	<0.001
Age (years)	62.71 ± 7.55	59.38 ± 5.72	60.73 ± 5.16	0.064
Current smoking, n (%)	292 (19.7)	153 (12.6)	50 (16.7)	<0.001
Drinking, n (%)	256 (17.3)	155 (12.8)	62 (20.7)	0.002
Sodium in taking, n (%)	305 (20.6)	197 (16.3)	56 (18.7)	0.003
Control of Diabetes, n (%)	81 (25.4)	55 (26.5)	19 (25.6)	0.189
Diabetes, n (%)	328 (22.1)	215 (18.8)	74 (20.1)	0.496
Use of ACE inhibitors or ARB, n (%)	87 (5.9)	54 (4.6)	18 (4.9)	0.635
Control of Hypertension, n (%)	415 (70)	252 (75)	68 (77)	<0.001
History of Hypertension, n (%)	594 (40.2)	337 (27.8)	90 (30.3)	<0.001
Waist circumference (cm)	85.04 ± 9.02	81.51 ± 8.93	84.29 ± 8.03	<0.001
BMI (kg/m2)	24.62 ± 3.03	24.27 ± 3.33	24.86 ± 3.08	0.001
SBP (mmHg)	141.12 ± 33.62	139.09 ± 20.14	140.16 ± 20.84	0.417
DBP (mmHg)	85.12 ± 28.46	83.42 ± 14.56	84.05 ± 14.98	0.179
FPG (mg/dl)	104.76 ± 26.46	102.58 ± 27.36	114.46 ± 37.98	0.001
HbA1c (%)	5.90 ± 0.87	5.90 ± 0.91	6.23 ± 1.23	0.001
TC (mmol/l)	5.21 ± 0.87	5.18 ± 0.85	5.06 ± 0.92	<0.001
TG (mmol/l)	2.14 (0.26,19.82)	1.89 (0.26,10.29)	1.84 (0.48,16.42)	<0.001
HDL-c (mmol/l)	1.38 ± 0.56	1.42 ± 0.50	1.42 ± 0.48	<0.001
LDL-c (mmol/l)	2.78 ± 0.73	2.71 ± 0.68	2.56 ± 0.67	<0.001
SUA (mg/dl)	5.22 ± 1.35	4.49 ± 1.16	3.95 ± 1.02	<0.001
BUN (mmol/l)	5.39 ± 1.30	4.99 ± 1.24	4.87 ± 1.27	<0.001
Serum creatinine (mg/dl)	0.89 ± 0.13	0.72 ± 0.09	0.60 ± 0.08	<0.001
eGFR (mL/min/1.73 m2)	78.44 ± 7.67	97.84 ± 3.23	106.27 ± 4.25	<0.001
MS components 0,1,2,3,4,5, (%)	7,24,26,26,14,3	13,25,26,20,13,3	12,26,27,21,12,2	<0.001

Data were presented as means (±SD) or number (%). Means and proportions were compared by ANOVA and Fisher’s exact test, respectively. *P* values testing the overall difference among eGFR Classifications. NGFR, normal eGFR; MRGFR, mildly reduced GFR; The eGFR Classifications of NGFR, MRGFR and hyperfiltration were in definitions. Definitions of hypertension, diabetes, MS were in methods. Sodium in taking; >6 g/day salt. *MS* metabolic syndrome, *BMI* body mass index, *SBP* systolic blood pressure, *DBP* diastolic blood pressure, *HDL* high density lipoprotein, *LDL* low density lipoprotein, *FPG* fasting plasma glucose, *TG* Triglycerides, *TC* Total cholesterol, *SUA* serum uric acid, *BUN*, blood urea nitrogen, *e-GFR* estimated glomerular filtration rate. International system of units (SI) conversion: plasma glucose 1 mg/dl = 1/18 mmol/l; SUA 1 mg/dl = 59.5 mmol/l; Serum creatinine, 1 mg/dL = 88.41umol/L

**Fig. 1:**
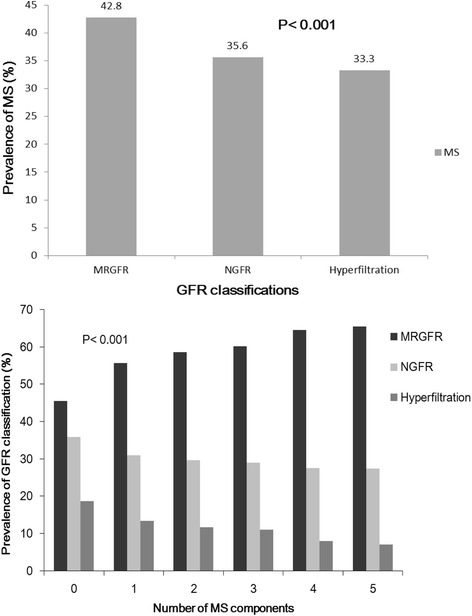
Relationship between metabolic syndrome (MS) and estimated glomerular filtration rate (eGFR) values. P refers to intergroup comparisons. Top panel: prevalence of MS according to eGFR classifications. The eGFR range for each classification is given in parentheses. Bottom panel: prevalence of eGFR classifications by number of MS components adjusted by sex and age (χ^2^ test). A normal eGFR (NGFR) was defined as ≥90 mL/min/1.73 m^2^. A mildly reduced eGFR (MRGFR) was defined as a value between 60 and 90 mL/min/1.73 m^2^

As shown in Fig. 1, the results revealed a significant, graded association between the number of MS components and a mildly reduced GFR (*P* < 0.001) for each comparison. Furthermore, there was a significant linear trend for the relationship between eGFR values and MS as a whole (top panel).

### Multiple linear regression analysis

As shown in Table 2, three models were constructed to analyze the association between different MS components and the eGFR value (HbA1c and FPG were analyzed separately due to co-linearity). The assumption of a linear relationship between the MS components and the eGFR value was assessed using a histogram of the residuals, together with a scatter plot of the standardized residuals to the standardized predicted value in the different models, showing an approximately linear relationship. In MS model, MS showed an independent, negative association with the eGFR value. However, after adjusting for age, gender, drinking and smoking status, hypertension, control of diabetes and hypertension and use of angiotensin-converting enzyme (ACE) inhibitors or angiotensin receptor blockers (ARBs), this negative association of MS and eGFR remained intact. In models 2 and 3, following multivariable adjustment, TG level and waist circumference were found to be negatively associated with the eGFR, whereas the FPG and HbA1c levels were positively associated with the eGFR.

**Table 2:** Multiple linear regression analysis of the relationship between MS components and eGFR

Models Independent variable	Unadjusted		Multivariable adjusted^a^	
	b Coefficient (95% CI)	*P*-value	b Coefficient (95% CI)	*P*-value
1 MS Model				
MS Y/N	-2.494 (−3.364 to −1.624)	<0.001	−1.329 (−2.098 to −0.520)	0.002
2 MS components Model				
SBP, per mmHg	0.003 (−0.013 to 0.019)	0.753	0.008 (−0.00 7to 0.022)	0.246
DBP, per mmHg	−0.008 (−0.028 to 0.012)	0.420	−0.012 (−0.029 to 0.005)	0.158
TG, per mmol/L	−1.082 (−1.414 to −0.750)	<0.001	−1.225 (−1.518 to −0.931)	<0.001
HDL, per mmol/L	0.360 (−0.444 to 1.165)	0.380	0.418 (−0.294 to 1.130)	0.250
Waist circumference, per cm	−0.310 (−0.369 to −0.252)	<0.001	−0.075 (−0.126 to −0.024)	0.004
BMI, per kg/m2	0.293 (0.128–0.458)	0.001	−0.043 (−0.195 to 0.109)	0.580
FPG, per mg/dL	0.443 (0.173–1.714)	0.001	0.740 (0.500–0.980)	0.024
3 MS components + HbA1C Model				
HbA1c, per % unit	0.978 (0.593–1.363)	<0.001	1.254 (0.850–1.657)	<0.001

*CI* confidence interval, *MS* Metabolic syndrome, *BMI* body mass index, *SBP* systolic blood pressure, *DBP* diastolic blood pressure, *HDL* high density lipoprotein, *FPG* fasting plasma glucose, *TG* Triglycerides, *e-GFR* estimated glomerular filtration rate. *HbA1c* Glycosylated hemoglobin A1c; ^a^Adjusting for age and gender, current smoking, history of hypertension, drinking, use of ACE inhibitors or ARB, control of diabetes and hypertension

### Multiple logistic regression analysis for mildly reduced eGFR

The unadjusted and multivariable-adjusted odds ratios for the association of a mildly reduced eGFR with the individual and multiple components of MS are reported in Table 3 for the four models. After adjusting for several potential confounders, the participants without MS showed a 1.29-fold increased odds ratio for a mildly reduced eGFR compared with those without MS. In comparison to participants without any MS components, there was a significant, stepwise increasing risk for the occurrence of a mildly reduced eGFR accompanying each additional component from 1 to 3 MS components. However, the risk associated with 4 and 5 MS components was lower than that associated with 3 MS components. In an attempt to explain this variation, the association between mildly reduced eGFR and individual MS traits was studied. Elevated TG, decreased HDL and obesity were significant risk factors of a mildly reduced eGFR in the multi-adjusted model, whereas elevated FPG levels reduced the risk of a mildly reduced eGFR. Furthermore, when elevated FPG levels were replaced by elevated HbA1c levels, similar results were obtained.

**Table 3:** Multiple logistic regression analyses of odds ratio for mildly reduced eGFR

Independent variable	Unadjusted		Multivariable adjusted^a^	
	OR (95% CI)	*P*-value	OR (95% CI)	*P*-value
1 MS Model				
MS Y/N	1.39 (1.20–1.61)	<0.001	1.29 (1.09–1.52)	0.003
2 Number of MS components Model				
0	1		1	
1	1.64 (1.25–2.16)	<0.001	1.38 (1.02–1.85)	0.035
2	1.85 (1.41–2.43)	<0.001	1.53 (1.14–2.06)	0.004
3	2.30 (1.74–3.03)	<0.001	1.94 (1.44–2.62)	<0.001
4	2.09 (1.54–2.84)	<0.001	1.58 (1.14–2.20)	0.007
5	2.03 (1.25–3.30)	0.004	1.53 (0.91–2.58)	0.111
3 MS components Model				
Elevated BP Y/N	1.21 (1.01–1.44)	0.040	1.02 (0.85–1.22)	0.853
Elevated TG Y/N	1.16 (1.00–1.35)	0.049	1.25 (1.05–1.49)	0.011
Decreased HDL Y/N	1.04 (0.84–1.29)	0.742	1.23 (1.03–1.48)	0.025
Obesity Y/N	1.42 (1.22–1.65)	<0.001	1.22 (1.03–1.45)	0.019
Elevated FPG Y/N	0.93 (0.77–1.11)	0.419	0.64 (0.52–0.78)	<0.001
4 MS components + HbA1C Model				
B1 (<6.5)	1		1	
B2 (≥6.5)	1.21 (1.03–1.42)	0.020	0.56 (0.45–0.70)	<0.001

*CI* confidence interval, *OR* odds ratio, *MS* Metabolic syndrome, *BP* blood pressure, *TG* Triglycerides, *HDL* high density lipoprotein; Obesity, a waist circumference > 90 cm in men and >80 cm in women; *FPG* fasting plasma glucose, *eGFR* estimated glomerular filtration rate; ^a^Adjusting for age and gender, current smoking, history of hypertension, drinking, use of ACE inhibitors or ARB, control of diabetes and hypertension

**Fig. 2:**
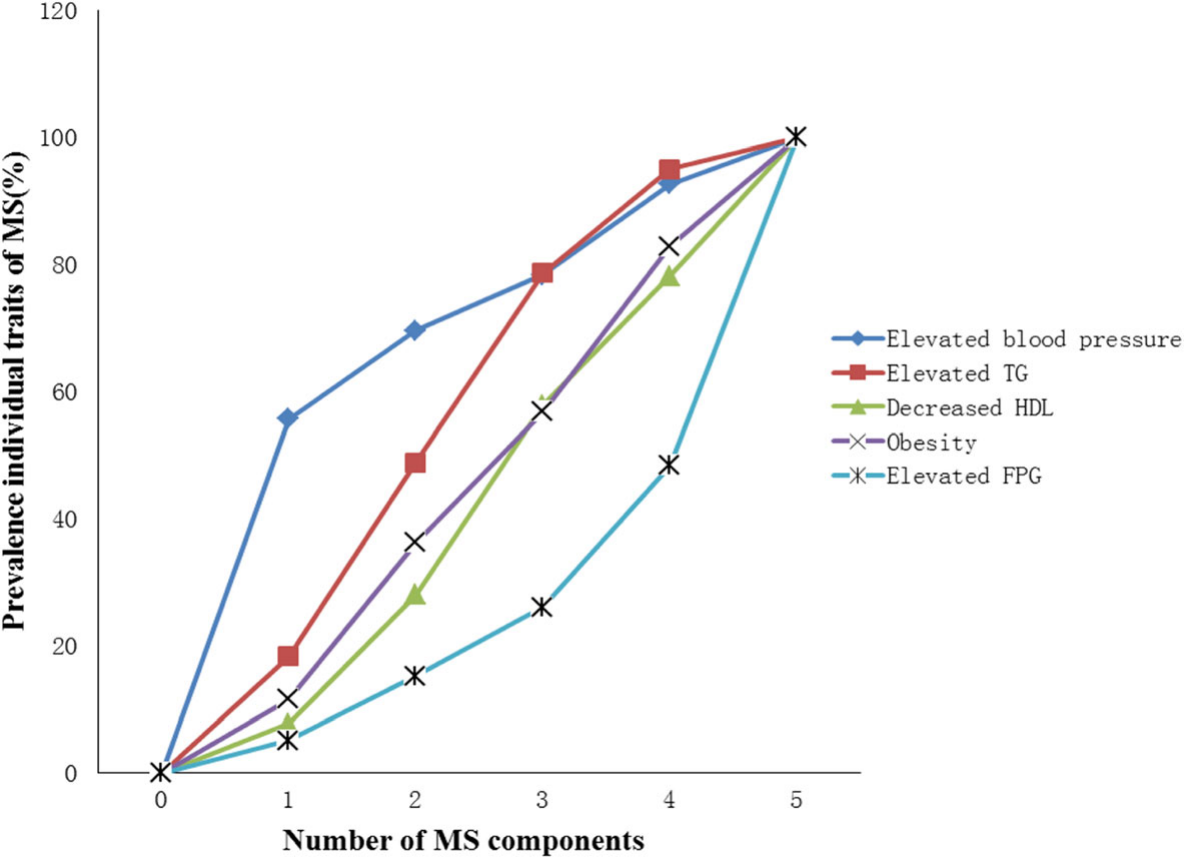
The variations of MS components’ prevalence according to the number of MS components. P refers to intergroup comparisons

Next, we investigated the variations of MS components’ prevalence according to the number of MS components (Fig. 2). When the number of metabolic components increased, the prevalence of individual MS traits gradually increased. In particular, the prevalence of elevated FPG levels in participants with 4 and 5 MS components was 48.4 and 100%, respectively, which was significantly higher increase than for the other components. Due to the different roles of certain MS components, participants with 5 MS components did not show an increased risk for a mildly reduced eGFR.

### Multiple logistic regression analysis for hyperfiltration

The 300 subjects (122 men) defined with hyperfiltration had mean GFRs of 103.27 (range 90.6–135.7) mL/min/1.73 m^2^. As shown in Table 4, we also analyzed the association between individual and multiple components of MS and an increased risk for hyperfiltration using the same four models. In MS model and number of MS components model, the presence of MS and the number of MS components were not associated with the hyperfiltration risk. However, after adjusting for age and gender, drinking and smoking status, hypertension, use of ACE inhibitors or ARBs and MS components (BMI, BP, TG, HDL-C), both elevated FPG and HbA1c levels showed an independent relationship with an increased risk for hyperfiltration.

**Table 4:** Multiple logistic regression analyses of odds ratio for hyperfiltration

Independent variable	Unadjusted		Multivariable adjusted^a^	
	OR (95% CI)	*P*-value	OR (95% CI)	*P*-value
1 MS Model				
MS Y/N	0.76 (0.60–0.96)	0.019	0.71 (0.71–1.21)	0.930
2 Number of MS components Model				
0	1		1	
1	0.80 (0.56–1.15)	0.223	1.06 (0.70–1.60)	0.798
2	0.62 (0.43–0.90)	0.011	0.81 (0.53–1.24)	0.326
3	0.66 (0.45–0.96)	0.029	0.95 (0.61–1.46)	0.798
4	0.44 (0.28–0.70)	0.001	0.73 (0.43–1.25)	0.249
5	0.51 (0.23–1.12)	0.094	0.89 (0.36–2.19)	0.797
3 MS components Model				
Elevated BP Y/N	0.82 (0.65–1.04)	0.100	0.98 (0.74–1.30)	0.844
Elevated TG Y/N	0.62 (0.49–0.79)	<0.001	0.56 (1.42–1.75)	<0.001
Decreased HDL Y/N	1.33 (1.04–1.70)	0.025	1.07 (1.81–1.42)	0.638
Obesity Y/N	0.69 (0.54–0.88)	0.002	0.98 (0.69–1.21)	0.527
Elevated FPG Y/N	1.05 (0.79–1.40)	0.717	1.53 (1.30–1.81)	<0.001
4 MS components + HbA1C Model				
B1 (<6.5)	1		1	
B2 (≥6.5)	1.50 (1.11–2.02)	0.008	2.86 (2.0–4.09)	0.000

*CI* confidence interval, *OR* odds ratio, *MS* Metabolic syndrome, *BP* blood pressure, *TG* Triglycerides, *HDL* high density lipoprotein, *Obesity* a waist circumference > 90 cm in men and >80 cm in women, *FPG* fasting plasma glucose; ^a^Adjusting for age and gender, current smoking, history of hypertension, drinking, use of ACE inhibitors or ARB, control of diabetes and hypertension

## Discussion

Cardiovascular mortality is approximately two-times higher in patients with stage 3 CKD (eGFR of 30–59 mL/min per 1.73 m^2^) and three-times higher in those with stage 4 CKD (15–29 mL/min per 1.73 m^2^) than in individuals with normal kidney function [20]. Even if a mild reduction in eGFR is observed, it is often accompanied by an increased risk for cardiovascular events, such as arterial stiffness, coronary artery calcium, myocardial hypertrophy, and even mortality [5, 6, 21]. MS may play an important role in mediating cardiovascular disease and the progression of CKD [22, 23]. In particular, two studies indicated that the rates of dyslipidemia and diastolic BP variability were significantly higher in patients with a mildly reduced eGFR than in participants with a normal eGFR [24, 25], suggesting that MS includes a series of metabolic abnormalities that may also be closely associated with a mildly reduced eGFR. Therefore, it is necessary to clarify the relationship between MS and a mildly reduced eGFR for the early diagnosis and preventive of renal damage.

In this Chinese population, which ranged in age from 40 to 79 years, we found that MS was independently associated with a mildly reduced eGFR following a multivariable adjustment. However, the participants with 1 to 4 MS components showed a significant, increased risk in the occurrence of a mildly reduced eGFR with the exception of participants with 5 MS components, compared with the participants without MS components, which is inconsistent with previous findings [13, 26–28]. In an attempt to explain this variation, we performed an in-depth analysis of the relationship between mildly reduced GFR and the various elements of MS, which showed that TG levels and waist circumference were negatively correlated with the eGFR following the multivariable adjustment, whereas the FPG and HbA1c levels were positively correlated with the eGFR. Furthermore, the multiple logistic regression analysis for mildly reduced eGFR indicated that elevated TG, decreased HDL and obesity significantly increased the risk for a mildly reduced eGFR in the multi-adjusted model, whereas elevated FPG and HbA1c levels presented the opposite effect. Accordingly, due to the dual roles of certain MS components, the presence of 5 MS components did not increase the risk for a mildly reduced eGFR in the models used in this study.

Several differences should be noted between our study and previous investigations [13, 24, 26–28]. First, the participants in this cross-sectional study were selected from a Chinese population, after excluding individuals with CHD, peripheral arterial disease, and CKD. However, Chinese individuals tend to have a lower GFR and a lower GFR rate of decrease compared with Western populations. Second, in our study, individual MS component showed different effects on the eGFR levels. Elevated TG, reduced HDL and obesity were independently associated with a mildly reduced eGFR, similar to other studies [13, 28]. However, elevated FPG (>6.11 mmol/L) and HbA1c levels (>6.5%) were independent factors for hyperfiltration, besides a reduced eGFR, which was similar to our early study [29]. Among the participants with diabetes, 58.8% were newly diagnosed and 41.2% had a diabetic history of 7.14 ± 6.02 years. The majority of the participants with abnormal blood glucose levels were in the pre-diabetes or early stages of diabetic nephropathy (DN), which are characterized by hyperfiltration (Additional file 1: Table S2). There are 1358 patients with pre-diabetes, accounting for 45.4% of the population. This finding may explain why participants with 5 MS components did not show an increased risk for a mildly reduced eGFR compared with participants without MS components in the present cross-sectional study. Several previous studies have shown that hypertension is a well-established risk factor for the progression of CKD [28, 30]. However, in our multivariable model, elevated BP was not associated with either a mildly reduced eGFR or hyperfiltration. This study focused on the associations between elevated BP and mildly reduced eGFR. Thus, participants with a GFR of 60–90 mL/min per 1.73m^2^ were recruited, and participants with a GFR less than 60 mL/min per 1.73m^2^ defined as CKD [13, 28] were not evaluated. Moreover, among the participant with hypertension, 47.3% were newly diagnosed and 52.7% had a hypertension history of 10.18 ± 7.54 years. The majority of the participants with hypertension were in the early stages of nephropathy. This findings may explain why elevated BP was not associated with either a mildly reduced eGFR or hyperfiltration in the present cross-sectional study. Third, we analyzed the relationship between MS and eGFR according to the presence of a mildly reduced eGFR and hyperfiltration, and we concluded that MS was independently associated with a mildly reduced eGFR. This finding is of great importance because CKD is associated with irreversible progression. Therefore, early detection of the contributing risk factors for a mildly reduced eGFR will be beneficial for the early prevention of CKD. Fourth, the definition of hyperfiltration varies between studies. The clinical relevance of hyperfiltration is based on a proposed pathologic effect of increased single-nephron GFR, which cannot be measured in humans. Moreover, the number of nephrons varies significantly between individuals, which is affected by many factors such as age, gender, weight, height and use of ACE inhibitors or ARB. Therefore, we chose a GFR value greater than the 90th percentile after adjusting for sex, age, weight, height and use of ACE inhibitors or ARB as indicative of hyperfiltration [16], rather than the arbitrary thresholds reported in previous studies, which range from 110 to 140 mL/min/1.73 m^2^ [31, 32].

MS has been linked to CKD in several cross-sectional studies [13, 26, 28]. However, the debate over the association between the individual components of MS and CKD or a reduced GFR has continued, which reveals our lack of recognition of the dynamic responses of the elements of MS during the development of renal dysfunction. The effect of MS on the process of CKD varies based on the presence and severity of different MS components as well as the nationality and race of the population under study. In our study, participants with an elevated FPG level only presented with hyperfiltration, whereas participants with elevated TG and/or reduced HDL and/or obesity showed a mildly reduced eGFR. In addition, participants with both elevated FPG and elevated TG and/or reduced HDL and/or obesity showed varying eGFR levels. Therefore, the eGFR levels cannot fully reflect the degree of renal damage. However, longitudinal studies on MS and CKD indicate that MS is linked to CKD [23, 33]. Hence, we inferred that both a mildly reduced eGFR and hyperfiltration increase the risk of CKD. MS had dual impact on renal damage. Nevertheless, it is necessary to perform follow-up studies on participants with mildly reduced eGFRs and hyperfiltration.

There were two significance of this decrease in renal function. First, many transverse and longitudinal researches indicated that mild renal function decrease would increase mortality from cardiovascular diseases [6, 34, 35]. However, mild renal dysfunction has not caused enough attention, and the recognition of its risk factors is insufficient. Researched about MS and severe renal dysfunction are adequate, while researches about MS and mild renal dysfunction are rare. Second, our previous study showed that hyperglycemia was the independent factor of hyperfiltration [29]. Thus, MS has double effects on GFR. On one hand, lipid, blood pressure and waist circumference decreased GFR. On the other hand, blood glucose increased GFR. Therefore, decrease of GFR might be severer than actual renal damages. MS patients with mild renal dysfunction should be paid attention to.

Our study had certain limitations. First, we chose the eGFR rather than the measured GFR, which is influenced by non-GFR factors, such as body composition and glycemic status [36]. Second, a cross-sectional study cannot infer the causality between MS, a mildly reduced eGFR and subsequent CKD. Therefore, longitudinal studies are needed to investigate whether MS factors associated with a mildly reduced eGFR represent risk factors for renal injury in the general population. Third, this study was a single-center study, and our population consisted primarily of urban workers who underwent an annual health checkup and had a low prevalence of CKD and severe DN.

## Conclusions

In conclusion, these results show that MS is associated with an increased risk for a mildly reduced eGFR in the middle-aged and elderly Chinese population. Moreover, individual components of MS play a different role on the eGFR; elevated TG, reduced HDL and obesity increase the risk for a mildly reduced eGFR, whereas elevated FPG and HbA1c levels increase the risk for hyperfiltration. Therefore MS had dual impact on renal injury. However longitudinal studies are needed to explore whether both a mildly reduced eGFR and hyperfiltration increase the risk for CKD in the Chinese population.

## Additional file

Additional file 1: Table S1. Usage of antihypertensive drugs and antihyperlipidimic agents. **Table S2**. GFR in patients with diabetes. (DOCX 15 kb)

## Abbreviations

ACE: Angiotensin-converting enzyme
ADA: American Diabetes Association
ARBs: Angiotensin receptor blockers
ATP III: Adult Treatment Panel III
BMI: Body mass index
BP: Blood pressure
BUN: Bblood urea nitrogen
CHD: Coronary heart disease
CIs: Confidence intervals
CKD: Chronic kidney disease
CKD-EPI: Chronic Kidney Disease Epidemiology Collaboration
DN: Diabetic nephropathy
eGFR: Estimated glomerular filtration rate
FPG: Fasting blood glucose
HDL-C: high-density lipoprotein-C
LDL-C: low-density lipoprotein-C
MRGFR: Mildly reduced eGFR
MS: Metabolic syndrome
NGFR: Normal eGFR
OGTT: Oral glucose tolerance test
PBG: Postprandial blood glucose
TC: Total cholesterol
TG: Triglycerides
