# Supplementary material for: Metabolic syndrome is independently associated with a mildly reduced estimated glomerular filtration rate: a cross-sectional study
Source: BMC Nephrol. 2017 Jun 13;18:192. doi: 10.1186/s12882-017-0597-3 (PMC5470228; doi:10.1186/s12882-017-0597-3)
Supplement: Additional file 1: Table S1. — Usage of antihypertensive drugs and antihyperlipidimic agents. Table S2. GFR in patients with diabetes. (DOCX 15 kb) [file 12882_2017_597_MOESM1_ESM.docx]

Table S1. Usage of antihypertensive drugs and antihyperlipidimic agents

| Characteristics | eGFR classifications | | | |
| --- | --- | --- | --- | --- |
|  | MRGFR (n=1479) | NGFR (n=1213) | Hyperfiltration (n=300) | P-value |
| Use of ACE inhibitors or ARB, n (%) | 87 (5.9) | 54 (4.6) | 18 (4.9) | 0.635 |
| Use of Diuretic, n (%) | 40 (2.7) | 25(2.1) | 7 (2.3) | 0.875 |
| Use of CCB, n (%) | 230(15.7) | 194(16.0) | 56 (18.6) | 0.546 |
| Use of Antihyperlipidimic agents, n (%) | 150 (10.1) | 109(9.1) | 32 (10.7) | 0.454 |

Notes: eGFR, estimated glomerular filtration rate; NGFR, normal eGFR; MRGFR, mildly reduced GFR; The eGFR Classifications of NGFR, MRGFR and hyperfiltration were in definitions. ARB: angiotensin receptor blocker; CCB: calcium channel blockers.

Table S2. GFR in patients with diabetes

|  | Control (n=1017) | Pre-diabetes (n=1358) | Diabetes (n=617) | P-value |
| --- | --- | --- | --- | --- |
| MRGFR n (%) | 468(46.0) | 683(50.3) | 328(53.2) | <0.001 |
| NGFR n (%) | 494(48.5) | 521(38.3) | 198(32.1) | <0.001 |
| Hyperfiltration n (%) | 55(5.4) | 154(11.3) | 91(14.7) | <0.001 |

Notes: NGFR, normal eGFR; MRGFR, mildly reduced GFR; The eGFR Classifications of NGFR, MRGFR and hyperfiltration were in definitions.
